# Functional ecology of planktonic ciliates: Measuring mortality rates in response to starvation

**DOI:** 10.1111/jeu.12969

**Published:** 2023-03-14

**Authors:** Thomas Weisse, Ulrike Scheffel, Peter Stadler

**Affiliations:** ^1^ Research Department for Limnology University of Innsbruck Mondsee Austria

**Keywords:** famine existence, FlowCAM, food limitation, imaging cytometry, laboratory experiments, loss rates, numerical response, protists, rate of decline

## Abstract

Population dynamics of aquatic ciliates are controlled “bottom‐up” via food supply and “top‐down” by grazing and parasitism. While intrinsic growth rates of ciliates under saturating food conditions have been studied in some detail, mortality rates induced by starvation have received little attention thus far. To this end, we examined the response of three algivorous freshwater ciliate species to starvation using three different optical methods. Two of these methods, i.e. ciliate mortality rates (*δ*) estimated from (i) numerical response experiments and (ii) the rate of decline (ROD) in cell numbers, investigated the response of the ciliate population using conventional light microscopy. The third method, imaging cytometry using a FlowCAM instrument, monitored single cells during the starvation experiment. Like light microscopy, the FlowCAM approach estimated *δ* based on ROD in the experimental containers. However, imaging cytometry also measured the relative cellular chlorophyll *a* content in the ciliates' food vacuoles as a proxy for the nutritional status of the cells. The linear decline of the cellular chl. *a* yielded an independent estimate of *δ* that was similar to *δ* calculated from ROD. Additionally, the FlowCAM measurements revealed a high degree of phenotypic plasticity of the ciliates when exposed to starvation.

## INTRODUCTION

Free‐living ciliates are significant players in planktonic food webs in the ocean and freshwater. Sitting at the interface between the classical grazer food chain and the microbial food web, they act as predators of pico‐ and nanoplanktonic bacteria and protists (mainly algae and heterotrophic flagellates) and food for microcrustaceans (copepods and cladocerans) and some fishes (Esteban & Fenchel, 2020; Lu & Weisse, 2022; Weisse & Montagnes, 2022). Ciliates are also important competitors of microcrustaceans and contribute significantly to nutrient regeneration in the pelagic realm (Caron, 1991; Caron & Goldman, 1988; Kamiyama, 2015). Like most aquatic protists, ciliates are subject to large fluctuations in their food supply and are adapted to a “feast and famine existence” (Fenchel, 1987, 1989; Poindexter, 1981). Most studies investigating the growth performance of planktonic ciliates focused on the “feast” part of their existence, i.e. maximum (or intrinsic) ciliate specific growth rates were recorded in relation to food supply and temperature under controlled laboratory conditions (reviewed by Montagnes & Berges, 2004; Weisse et al., 2016). However, due to food limitation, intrinsic growth rates grossly overestimate the ciliates' performance under most natural conditions (Fenchel, 1987; Gaedke & Straile, 1994; Lu et al., 2021). In the nutrient‐deplete central gyres of the oceans and many (ultra)oligotrophic lakes where food is scarce, ciliates can only survive if their threshold prey level (i.e. the food abundance where growth is zero, Montagnes, 1996; Montagnes & Berges, 2004) is low. Cyst formation, an alternative strategy to survive unfavorable environmental conditions, including starvation (Gutiérrez et al., 2001; Kamiyama, 2013; Verni & Rosati, 2011), is not an option in permanently food‐limited, deep‐water environments.

As yet, three principal methods are available to measure the ciliates' response to starvation: (1) Numerical response (NR) experiments can parameterize the threshold prey density, i.e. assessing growth rates as a function of food levels (reviewed by Montagnes & Berges, 2004; Weisse et al., 2016). NR experiments are fitted to a rectangular hyperbolic function (Equation 2, below) that can be used to predict food‐dependent ciliate growth and mortality rates (Minter et al., 2011; Montagnes & Berges, 2004; Weisse et al., 2016). Importantly, in contrast to older assumptions (Fenchel, 1987), mortality is not constant but varies with prey density (Li & Montagnes, 2015). From Equation 1, mortality rates can be predicted at zero food levels. Such calculations provide a theoretical estimate of maximum mortality because aquatic protists rarely meet natural conditions devoid of food. A more pragmatic approach to determining mortality rates is (2) to measure the rate of decline (ROD) of a protist population at low to nearly zero food levels (e.g. Jackson & Berger, 1984, 1985; Minter et al., 2011). In the present study, we use both approaches to ascertain the mortality rates of pelagic ciliates. We complement these indirect measurements targeting the population level by (3) analyses of single cells exposed to starvation. To this end, several features characterizing the nutritional status of ciliates were measured by an imaging cytometer (FlowCAM, Sieracki et al., 1998) and images of measured cells were recorded for further analyses. Up to now, the FlowCAM method has been mainly applied for phytoplankton analysis (Buskey & Hyatt, 2006; Hrycik et al., 2019; Poulton, 2016) and to assess plankton size spectra (Álvarez et al., 2011, 2014). Our approach to measuring the nutritional status of single cells is novel for heterotrophic protists. Combining single‐cell measurements with those at the population level is instrumental for functional ecology analysis and can be applied to many other heterotrophic and autotropic aquatic protists.

This report is the first of several articles investigating the effects of starvation on planktonic ciliates in response to food and temperature. We use ciliates as model organisms because they are significant in aquatic food webs (see above) and can be experimentally manipulated with ease. A follow‐up study will present several case studies of starvation experiments with common freshwater ciliates using the methods given in the current work (Weisse et al., 2023). Finally, we will use a meta‐analysis of the literature to explore potential differences between freshwater and marine ciliates and to predict their response to high temperatures, which is essential in the ongoing global warming (T. Weisse, unpublished data).

## MATERIALS AND METHODS

### Mortality rates inferred from NR

The theoretical background and general experimental design of NR experiments have been reviewed recently (Li et al., 2013; Montagnes, 2013; Weisse et al., 2016) and shall not be repeated here. We used unpublished material with the freshwater ciliate *Pelagostrombidium mirabile* (Penard, 1916) Krainer, 1991 as an example to illustrate the experimental approach and calculation of mortality rates.

The ciliate was isolated from the epilimnion of oligomesotrophic Lake Mondsee (Austria) in spring 2021. Clonal cultures were obtained from enrichment cultures using the cryptophyte *Cryptomonas* sp. strain 26.80 provided by the Culture Collection of Algae in Göttingen (Germany) as food. This flagellate, which provides excellent food for ciliates and other freshwater microzooplankton (Lu et al., 2021 and citations therein; Weisse & Frahm, 2002), is approximately 11 μm in length. Similar to previous studies in our laboratory (e.g. Lu et al., 2021; Weisse et al., 2002), the cell volume of *Cryptomonas* sp. (~290 μm^3^) was measured by an electronic particle analyzer (CASY 1‐model TTC; Schärfe System).

Ciliate stock cultures were kept in culture flasks (50 mL volume) with *Cryptomonas* sp. at constant light conditions (100 μmol photons m^−2^ s^−1^) and 15°C. Experiments were conducted at moderate light levels (30 μmol photons m^−2^ s^−1^, 12:12 light: dark cycle) over food abundances ranging from zero to ~90,000 *Cryptomonas* sp. cells mL^−1^. The ciliates were gradually acclimated to the experimental prey levels over several days. The final adaptation period at the experimental target food levels lasted for 24 h.

Experiments were conducted in six‐well culture plates (well volume = 10 mL) for 24 h. Subsamples for determining ciliate and food prey abundances were taken at the beginning and end of the experiments and fixed with acid Lugol's solution. Cell numbers of predators and prey were counted microscopically in sediment chambers of 3‐mL volume (ciliates), respectively, in Sedgwick rafter cells of 1‐mL volume (*Cryptomonas* sp.).

Ciliate growth rates were calculated from the change in cell numbers according to

(1) $$r=\ln\;\left(N_{t}/N_{0}\right)/t$$

where *N*_0_ denotes the initial and *N*_*t*_ the final ciliate abundance (cells mL^−1^); *t* is the experimental duration (d).

The NR was calculated from Equation 2:

(2) $$r=r_{\max}\;\left(P–P^{\prime }\right)/k+\left(P–P^{\prime }\right)$$

*r*_max_ is the maximum (or intrinsic) specific growth rate (d^−1^), *P* is the mean prey concentration (cells mL^−1^) during the experiments, *k* is a constant (cells mL^−1^), and *P′* is the *x*‐axis intercept (i.e. the threshold concentration, where *r* = 0). Nonlinear curve fitting was performed in SigmaPlot for Windows (version 14.5.0.101) using the Marquardt–Levenberg algorithm that provides asymptotic standard errors for the parameters of Equation 2 (Montagnes & Berges, 2004).

The geometric mean prey concentration (*P*) was determined as.

(3) $$P=\left(P_{t}–P_{0}\right)/\ln\;\left(P_{t}–P_{0}\right)$$

*P*_0_ and *P*_*t*_ are the initial and final food concentrations (cells mL^−1^).

To express prey levels as biomass, we converted the cell volume (in μm^3^) of *Cryptomonas* sp. to carbon units (pg C cell ^−1^), assuming C = 0.261 × volume^0.860^ (Menden‐Deuer & Lessard, 2000). Accordingly, an average *Cryptomonas* sp. cell contained 34 pg C.

Ciliate mortality rates (*δ*, day^−1^) were calculated from Equation 2, setting *P* to zero (i.e. no food).

### Mortality rates measured from the ROD

The *Meseres corlissi* Petz & Foissner, 1992 clone AUS used in this study had been sampled from the floodplain of the Murray River, a warm‐temperate environment in SE Australia in March 2006 (Gächter & Weisse, 2006). The cultures were obtained from excysted cells, according to Foissner et al. (2002). Stock clonal cultures of *M. corlissi* were fed with the same *Cryptomonas* sp. strain used for the experiments with *P. mirabile* and kept in modified Woods Hole Medium UKNCC, 2001 enriched with 10% soil extract (Müller et al., 2006) as described previously (Gächter & Weisse, 2006; Weisse, 2004). Stock cultures were kept in tissue culture flasks of 50 mL volume at 22.5°C and light intensity of ~50–70 μmol photons m^−2^ under a 14:10 h light: dark cycle.

The ciliates were stepwise acclimated to the experimental temperature of 25°C over 3 d before the beginning of the experiments. The experimental duration was 48 h. The ciliates were fed with algae for the last time 3 d before the beginning of the experiments. At the end of the acclimation period, the algal abundance was <10,000 cells mL^−1^, i.e. ciliates were moderately starved. An aliquot (~5 mL) of the acclimated culture was poured into a small Petri dish (5 cm diameter), and ~200 ciliates were pipetted out and transferred to a second Petri dish containing MWC medium and Volvic table water in a ratio of 1:1, but no algae. This step was repeated, i.e. ciliates were washed twice before 15 ciliates each were pipetted into the first three wells of a 12‐well tissue plate, filled with 4 mL each of the MWC medium and Volvic table water suspension. Another ten ciliates each were pipetted in the first three wells of the second row (B1–B3) of the same 12‐well tissue plate, filled with 4 mL each of the MWC medium and Volvic table water suspension plus *Cryptomonas* sp. at satiating food levels (~90,000 cells mL^−1^). This treatment served as a control to rule out that ciliate abundance decreased due to factors other than depletion of food. Ciliate cell numbers in all wells of the tissue plate were counted under a dissecting microscope twice day^−1^ during the experiment. To prevent algal growth, the experiments were conducted in the dark.

Growth rates (*r*, d^−1^) and mortality rates (*δ*, d^−1^) of *M. corlissi* were calculated from the slope of the change in logarithmized cell numbers over time in each experimental container. Since mortality represents negative growth rates (i.e. the slope of the least‐squares linear regression is negative), we multiplied *δ* by −1 to report positive values in the following.

### Mortality rates inferred from imaging cytometry

We used an imaging flow cytometer (FlowCAM®, Fluid Imaging Technology) for counting live cells and analyzing several morphological and physiological properties of single cells of the common mixotrophic freshwater ciliate *Coleps spetai* Foissner, 1984 in response to starvation. The FlowCAM is a combination of a flow cytometer and a microscope with a digital camera attached that characterizes each cell within a sample with ~30 morphological and fluorescence parameters (Sieracki et al., 1998). FlowCAM measurements of 1 mL volume each were conducted in two modes: (i) AutoImage Mode captures images of fluorescent and nonfluorescent particles in the sample fluid; (ii) Trigger Mode uses the green laser installed (532 nm) to excite fluorescent particles characterized by their cellular chlorophyll *a* and accessory pigments such as phycoerythrin. Images of measured cells were analyzed using the Visual Spreadsheet software (Version 3.7.5). Each measurement was performed in one or two runs. Therefore, the total volume of the subsamples taken for the FlowCAM analyses was 5 mL. For further details, consult Bergkemper and Weisse (2018).

*Coleps spetai* was isolated from oligo‐mesotrophic Lake Mondsee, Austria (Weisse & Rammer, 2006). Like *P. mirabile* and *M. corlissi*, clonal cultures of *C. spetai* were maintained with *Cryptomonas* sp. strain 26.80 as food in modified Woods Hole Medium. The ciliates were moderately starved before the beginning of the experiments. At the beginning, the ciliates were washed three times in sterile filtered sterile‐filtered water from Lake Mondsee and pipetted into the experimental containers. The subsampling every 24 h required a larger container volume than the tissue‐plate experiment reported above. Therefore, we used 50‐mL culture flasks for *C. spetai*. The experiments were run at 25°C in the dark to rule out that *C. spetai* benefitted from photosynthesis of its endosymbionts. Initial ciliate abundance was ~190 cells mL^−1^ in the culture flasks and ~140 cells mL^−1^ in the controls. The experimental treatments received no food; the controls were run at saturating food concentrations (~48,000 *Cryptomonas* sp. cells mL^−1^, equivalent to ~1.6 μg C mL^−1^) in the dark. The experiments lasted for 3 d. All experiments and controls were run in triplicate. Ciliate and flagellate cell numbers were counted in live subsamples of 0.25–1 mL volume each. Cell numbers of *Cryptomonas* sp. were also measured in Lugol's‐fixed material as described above.

## RESULTS

### Mortality rates of *Pelagostrombidium mirabile* inferred from NR

**FIGURE 1:**
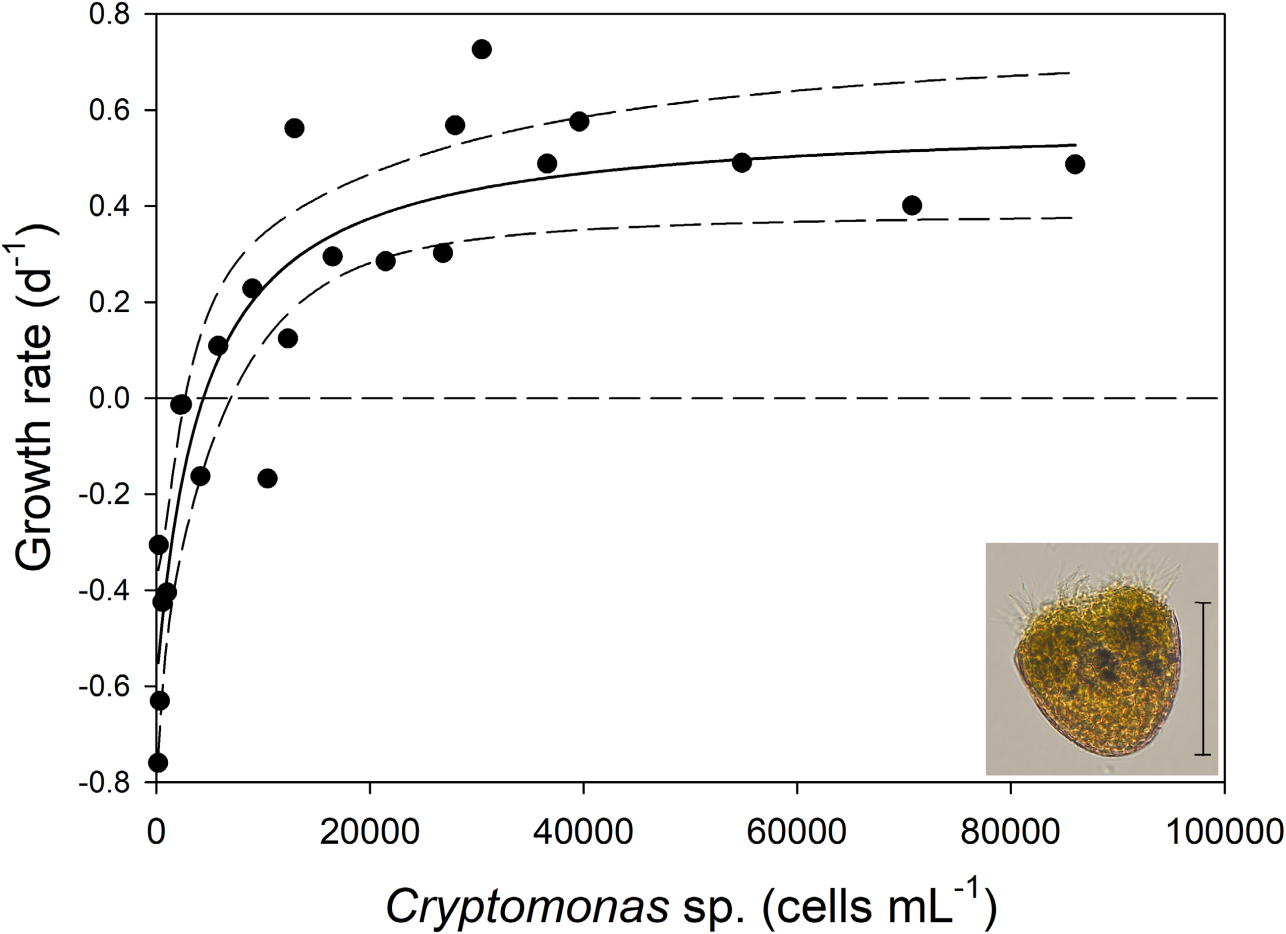
Numerical response of the ciliate *Pelagostrombidium mirabile* fed with the flagellate *Cryptomonas* sp. The insert shows a Lugol's fixed ciliate cell (scale bar = 40 μm).

The NR of *Pelagostrombidium mirabile* followed the typical rectangular hyperbolic function (Equation 2; Figure 1). The maximum specific growth rate, *r*_max_, was 0.58 ± 0.09 d^−1^. The threshold concentration (*P′*) was reached at 4390 ± 1057 *Cryptomonas* sp. cells mL^−1^, corresponding to 0.15 ± 0.04 μg C mL^−1^. The constant *k* was 8665 ± 2877 *Cryptomonas* sp. cells mL^−1^, equivalent to 0.29 ± 0.10 μg C mL^−1^. The fit of the NR curve (*R* ^2^ = 0.841) and all model parameters were highly significant (*p* < 0.01), thus allowing relatively precise mortality rate calculation. Without any food (*P* = 0 in Equation 2), the *P. mirabile* population would decline at a rate of 0.58 d^−1^ (0.36–0.79 d^−1^, range of 95% confidence interval) at the experimental temperature of 15°C. The cell size of Lugol's fixed *P. mirabile* was approximately 45 × 38 μm.

### Mortality rates of two ciliate species inferred from the ROD

**FIGURE 2:**
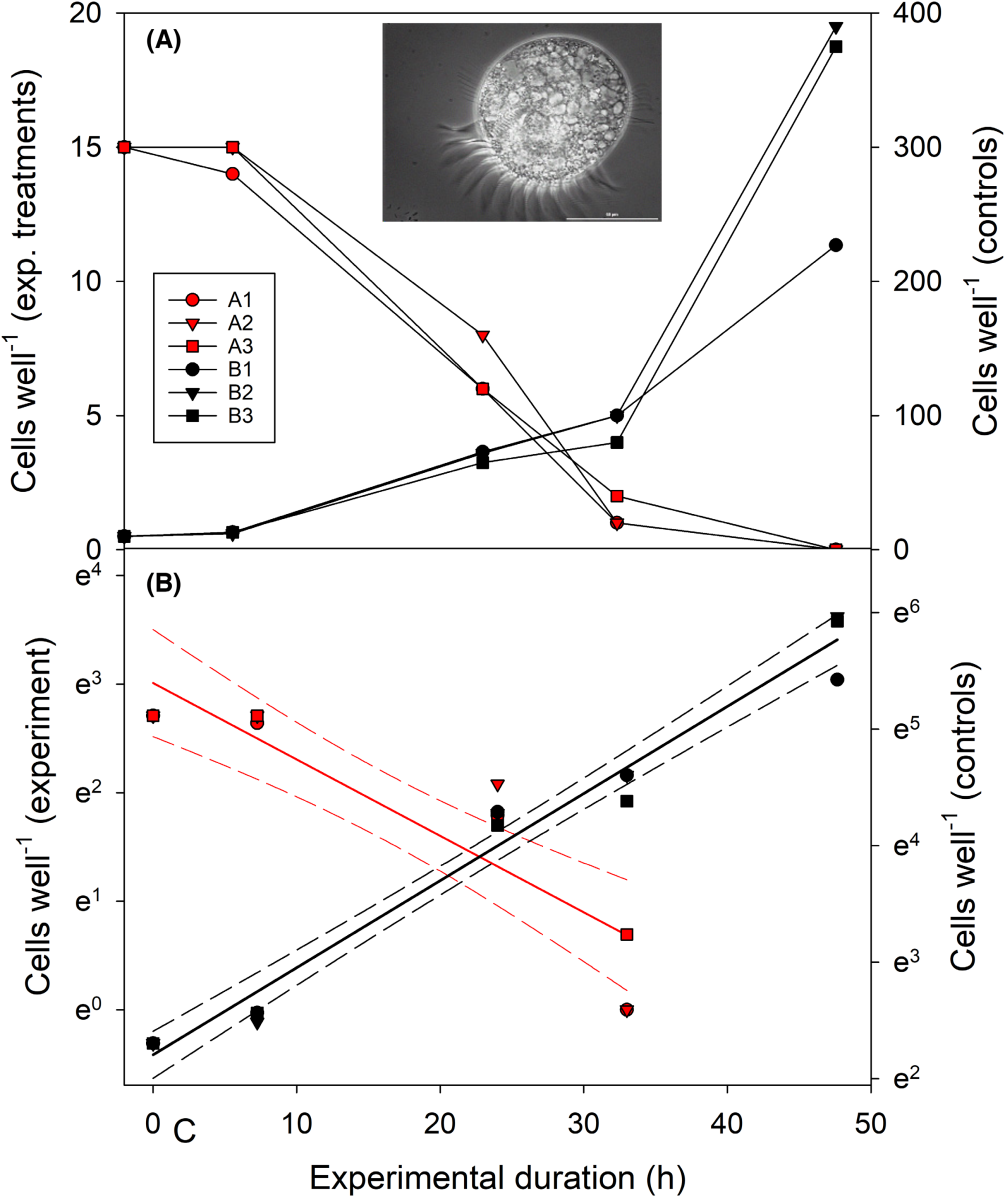
(A) Change in cell numbers of *Meseres corlissi* in the starved experimental treatments (red symbols) and in controls (black symbols) kept at saturating food conditions (~90,000 *Cryptomonas* sp. cells mL^−1^). No ciliate cells occurred in all three experimental replicates after 48 h. (B) The slope of the linear regressions of ln cell numbers versus time (solid lines) denotes the ciliates' growth rate (*μ*, h^−1^) in the controls (black symbols) and the mortality rate (*δ*, h^−1^) of *M. corlissi* in the experimental treatments (red symbols). Dashed lines indicate 95% confidence intervals. Note that in both plots, several symbols overlap. The microphotograph in A shows a live *M. corlissi* cell under phase contrast.

**FIGURE 3:**
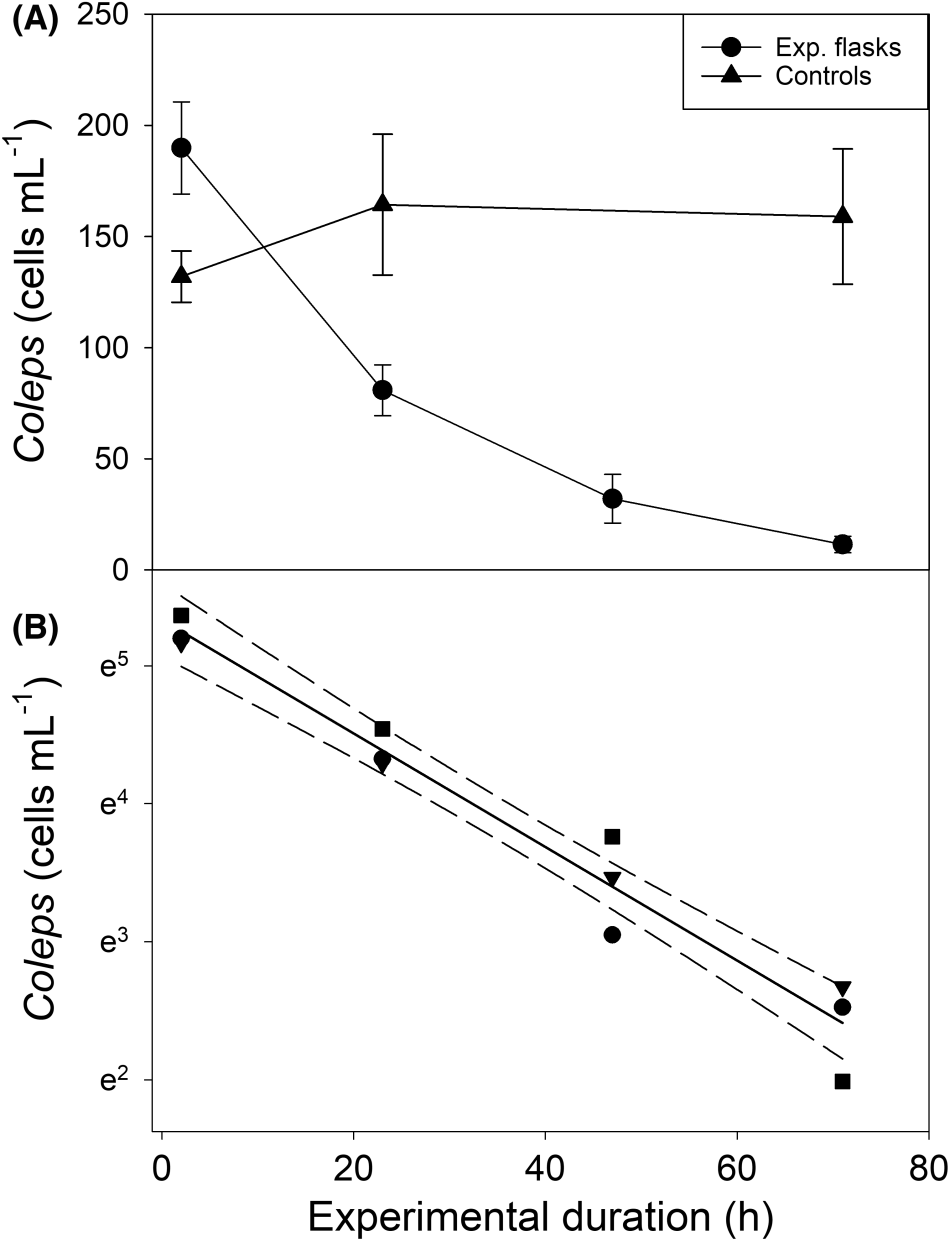
(A) Change in cell numbers of *Coleps spetai* in the starved experimental treatments and in controls kept at saturating food conditions (~48,000 *Cryptomonas* sp. cells mL^−1^). Symbols represent mean values of three replicates each; the error bars denote standard deviation (SD). (B) Linear regression (solid line) with 95% confidence intervals (dashed lines) of *C. spetai* cell numbers versus time in the experimental treatments (three replicates each). Ciliate cells were measured alive by FlowCAM.

We measured the ROD of a ciliate population using fixed material and traditional light microscopy (Figure 2) or automated optical counting techniques of live ciliate cells (Figure 3). The experimental temperature was 25°C in both case studies.

The cell numbers of *Meseres corlissi* in the 12‐well tissue plate declined in the experimental treatments and increased exponentially in the controls (Figure 2A). Ciliate population development was similar between the replicates of the experimental, respectively the control treatments. The linear regressions of ln cell numbers versus time yielded a specific growth rate (*r*) of 1.79 ± 0.08 d^−1^ and a mortality rate (*δ*) of 1.69 ± 0.26 d^−1^. Both regression coefficients were significant (*p* < 0.01). The initial cell length of Lugol's fixed *M. corlissi* was 50.8 ± 6.0 μm.

**FIGURE 4:**
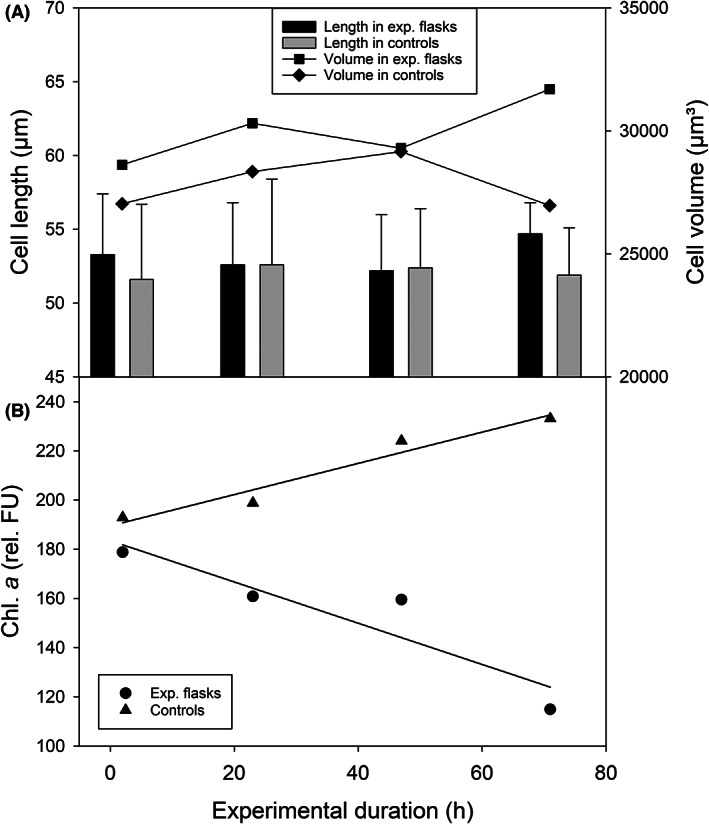
(A) Change in cell length (bar plot) and cell volume (line plot) and (B) relative chl. *a* content (Fluorescence Units) of *Coleps spetai* during the starvation experiment shown in Figure 3. The error bars in A denote SD of the cell length. For clarity, we do not show the SD for cell volume (in A) and confidence intervals for the relative chl. *a* content (in B).

Figure 3 shows the decline of a population of *Coleps spetai* upon starvation measured by imaging flow cytometry (FlowCAM). The slope of the linear regression of ln cell numbers versus time in the experimental flasks was −0.041 ± 0.003 h^−1^ (*R* ^2^ = 0.959), i.e. the mortality rate of *C. spetai* was 0.99 ± 0.06 d^−1^ at 25°C. The initial cell length of live *C. spetai* was 53.3 ± 4.1 μm (Figure 4A).

### Monitoring single cells by imaging cytometry during starvation

We monitored ten morphological (e.g. cell length, width, volume, circularity) and several physiological features of *Coleps spetai* during the starvation experiment shown in Figure 3. First, the morphological variables showed no trend with increasing duration of starvation (Figure 4A). Secondly, there was no difference between the experimental treatments without food and the controls with food (paired *t*‐tests). The coefficient of variation (CV) amounted to, on average, 7.2% in cell length and 20.2% in cell volume, i.e. there was some phenotypic plasticity in cell morphology, but there was no difference between starved and well‐fed ciliates.

The relative chlorophyll *a* content of the *Coleps* cells is a proxy for the prey (*Cryptomonas* sp.) content in their food vacuoles and pigments originating from the endosymbionts of *C. spetai*. The cellular chl. *a* content declined linearly with the period of starvation but increased in the controls with food (Figure 4B). From the slope (a) of the linear regression shown in Figure 4B (*a* = −0.839 h^−1^, *R* ^2^ = 0.847), we can calculate that it would take 219 h (9.1 d) until no more chl. *a* can be detected in the ciliate population. In the controls, the mean CV of the cellular chl. *a* was relatively constant, ranging from 26.4% (recorded on day 3 after the beginning, i.e. at the end of the experiments) to 34.6% (on day 1). In the experimental containers, the mean CV of cellular chl. *a* increased from 38.5% at the beginning to 76.1% at the end of the experiment.

**FIGURE 5:**
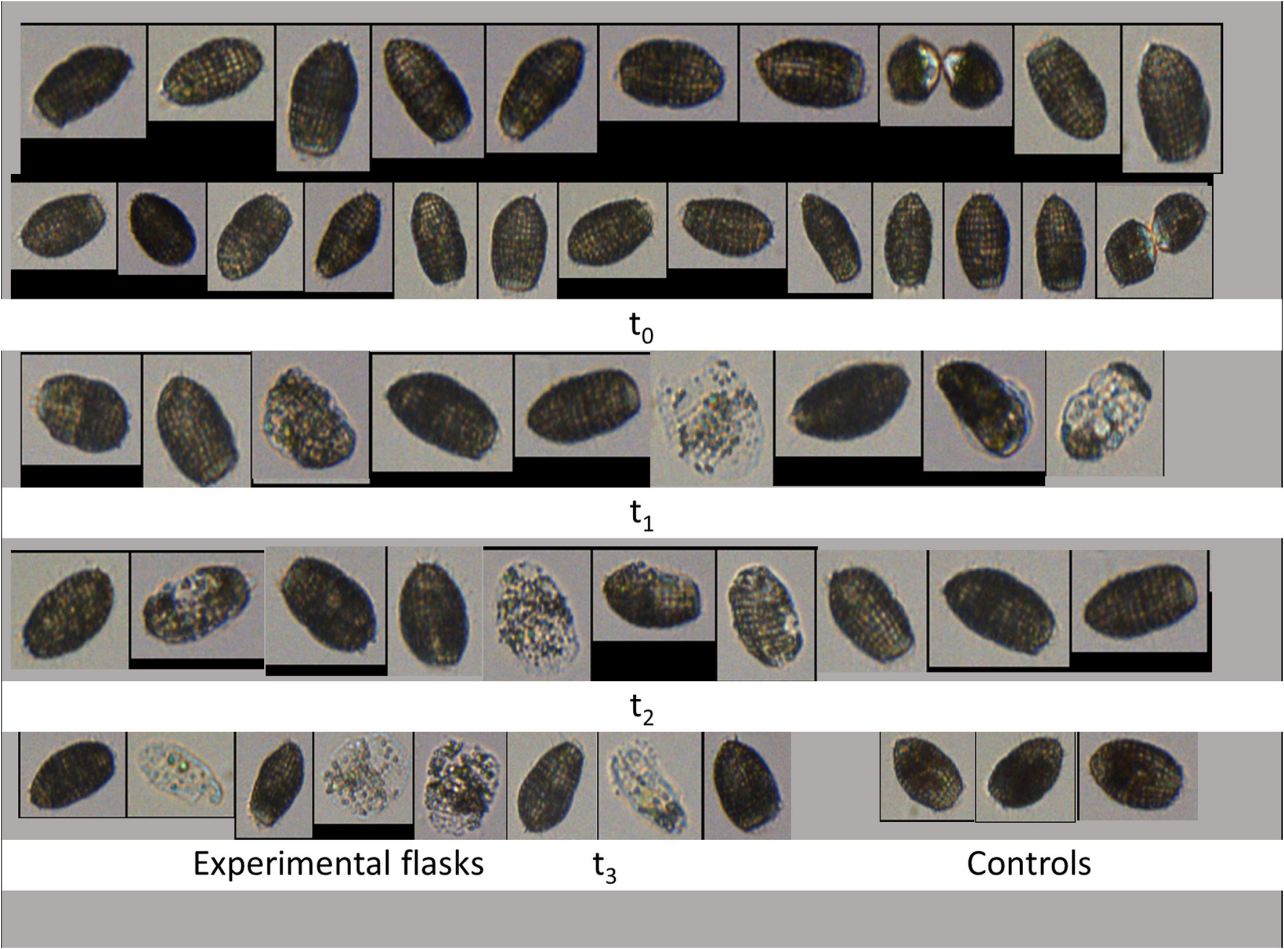
FlowCAM images of *Coleps spetai* during the starvation experiment reported in Figures 3 and 4. The time (*t*) denotes the experimental duration (in d), counted from the beginning (*t*_0_). Cells in the controls are shown at the beginning (*t*_0_, second row) and the end of the experiment (*t*_3_, bottom right).

The FlowCAM images taken during the experiments illustrate the deterioration of the *Coleps* cells with increasing time of starvation (Figure 5). At the beginning, all cells looked healthy, both in the controls and in the experimental flasks without food, and dividing cells and newly dividers were visible in both treatments (Figure 5, top two rows). The first obviously damaged cells occurred in the experimental flasks one day after starvation onset, and the endosymbionts became visible. The empty or burst cells' proportion increased on days 2 and 3. At the end of the experiment, approximately 50% of the starved ciliates appeared dead in the experimental treatment, i.e. only the calcified armored plates (Foissner et al., 1999), a few endosymbionts and some remains of the internal cell structure were visible (Figure 5). However, other cells looked healthy and seemed unaffected by starvation. Accordingly, the visual inspection of the cells was in line with their physiological status obtained from their relative chl. *a* content, showing considerable individual variation within the population at the end of the experiment.

## DISCUSSION

Parameterizing mortality rates (*δ*) of planktonic ciliates and other protists is crucial for food web models aiming to predict the protists' response to changing environmental conditions. For instance, rising water temperatures caused by ongoing global warming may lead to higher food thresholds (Weisse et al., 2002), thereby enhancing the mortality of heterotrophic protists at low food conditions. Although the NR is instrumental in analyzing the relation between specific growth rates and food, this is (i) only available for a tiny percentage of planktonic protist species and, as we will discuss below, (ii) not always straightforward. Estimates of mortality rates from changes in population densities *in situ*, available with several modifications (Minter et al., 2011), represent rather net loss rates than mortality rates and are often case‐specific. Alternative methods are therefore needed to verify and complement results obtained from NR studies. To this end, we present in this study two alternative ways to determine ciliate mortality rates and discuss the pros and cons of each approach in the following.

### Mortality rates inferred from NR

The nonlinear regression shown in Figure 1 yielded high goodness of fit, with all three NR parameters (Equation 2) being significant. The large majority of the data fell within the 95% confidence interval, i.e. the growth response of *Pelagostrombidium mirabile* can be accurately predicted as a function of prey supply. This is often not the case; first, several NR studies did report (if at all) only the overall curve fit (*R* ^2^) but not the significance levels or error terms of the parameter estimates (e.g. Jakobsen & Hansen, 1997; Smith & Hansen, 2007). Secondly, the statistical power of asymptotic error terms from nonlinear fits is lower than that of error terms obtained from linear regressions (Montagnes & Berges, 2004). However, accurate estimates of *k* and *P′* are needed to calculate mortality rates (=negative growth rates) at zero food levels (i.e. *P* = 0). This is problematic if the initial slope of the NR curve (*α*, see Weisse et al., 2016) is very steep. In such a case, Equation 2 yields positive mortality rates if *k* < *P′* (e.g. for the marine oligotrich ciliate *Strombidium capitatum*, Montagnes, 1996) or, if *k* is only slightly higher than *P′*, the asymptotic approximation will yield unrealistically high estimates of *δ*. For instance, for a *Meseres corlissi* clone isolated from Central America, the parameters of the NR (*r*_max_ = 2.82 ± 0.11 d^−1^, *P* = 0.10 ± 0.01, *k* = 0.13 ± 0.02) measured at 25°C (Weisse, 2004) yield *δ* = 9.4 d^−1^. Even considering that this was a different *Meseres* clone as used in the present study, our considerably lower estimate provided above (*δ* = 1.69 ± 0.26 d^−1^) from ROD appears more realistic. This conclusion is evident if we compare the calculated mortality rate to the measured growth rates at food levels below the threshold (< *P′*), which were close to −1.2 d^−1^ (Weisse, 2004). In general, predictions of *δ* derived from NR experiments appear unrealistic if they fall clearly (i.e. by more than a factor of 2) outside of the observed values that were used to fit the regression model.

In conclusion, if the initial slope of the NR curve is very steep, *δ* should not be calculated from the parameters of the nonlinear function but estimated from the negative growth rates measured at the lowest food concentrations tested. The measured values may slightly but not dramatically underestimate the “true” value of *δ*.

### Mortality rates inferred from the ROD

Estimating mortality rates from a protist population's ROD is straightforward but requires that food is absent in the experimental containers. This assumption is difficult to meet since most experiments with planktonic protists are conducted under nonsterile conditions. Bacteria, which are usually present in cultures of heterotrophic (“algivorous” or “omnivorous”) protists, may benefit from substrates supplied with the culture medium. Accordingly, they may increase their standing stock in the course of the experiment, in particular, if there is no competition for nutrients with algae. Filter‐feeding protists, which prefer motile algal prey such as *Cryptomonas* under natural conditions, may take up bacteria under food‐deplete conditions. Even if the latter are suboptimal food, they may alleviate the starvation of the protists. Secondly, it is difficult to avoid contamination of the starvation containers with algae during the inoculation process. We monitored the presence of algae and bacteria in the experimental vessels microscopically and by flow cytometry during the starvation experiments. In the case studies presented here, the algal and bacterial background remained low during the experiments (e.g. 2–5 *Cryptomonas* cells mL^−1^ in the experiment with *Coleps spetai* and bacterial levels ranging from (0.3–0.8)*10^6^ mL^−1^). If imaging flow cytometry is available, algal contamination can be directly assessed in the experimental containers.

The bias resulting from bacterial contamination tends to increase with experimental duration. If bacteria are used as food by the target protists, the exponential decline of the protist population may become lower after several days. This effect will become visible in the plot of ln cell numbers versus time by deviation from the linear regression calculated for the initial days of the experiment. In conclusion, contamination with potential food is more severe in ROD experiments running over several days than in NR experiments that are usually short‐termed (~1 d). Analytical and statistical tools provide a handle to estimate the potential bias originating from “hidden food” for calculating mortality rates.

Some caution is needed in ROD experiments with cyst‐forming ciliates such as *Meseres corlissi*. Since food depletion may trigger encystment (Kamiyama, 2013; Müller, 2000), the ROD may disproportionately increase several days after the beginning of the experiment. In another experiment using larger containers (flasks of 50‐mL volume), which we conducted in parallel to the “well” experiment shown in Figure 2, mass encystment was recorded 48 and 72 h after the beginning of the experiment (Weisse et al., 2023). Cysts were also present in several wells of the current studies after 48 h. To this end, ROD was calculated only for the initial 33 h of the well experiment (Figure 2B) when encysted cells contributed <5% to the *Meseres* population.

### Using single‐cell analysis for estimating mortality rates

The methods discussed in the previous sections yield the average mortality of a protist population in response to starvation, which is a valuable parameter for analyzing population dynamics in food‐web or predator–prey models (Minter et al., 2011) and an inherent feature of a “species” functional ecology. However, even if clonal protist cultures are used, there is always intraspecific variability (Weisse & Rammer, 2006; Yang et al., 2013). Between‐individual variation in physiological traits within nonclonal and clonal populations is the rule rather than the exception in most eukaryote populations (reviewed by Spicer & Gaston, 2009). From an evolutionary point of view, the best and worst performers in a population are more important than the population mean. Analyzing single cells in a protist population allows calculating the individual variation in response to starvation. Applying imaging flow cytometry in the case study with *Coleps spetai* demonstrated that even in a heavily starved population, some cells appear virtually unaffected by the depletion of food. In a follow‐up study to the present work, we present more details on the endosymbionts of *C. spetai* and suggest that the ciliate may have benefitted from resources provided by its endosymbionts (Weisse et al., 2023).

We used the relative cellular chl. *a* content as a proxy for the nutritional status of the cells. From the linear ROD of the cellular chl. *a* (Figure 4B), we estimated that no food remained in the ciliates' food vacuoles after 9.1 days. This result is remarkably similar to our estimate of mortality (δ = 0.99 d^−1^, Figure 3B) derived from the exponential decline of the population; the latter estimate implies that after nine days of starvation, the *Coleps* population would have been reduced to <0.03 cells mL^−1^.

## CONCLUSIONS

First, the three methods we presented above yielded consistent estimates of ciliate mortality rates (*δ*). If converted to positive values, *δ* were similar to the respective species' maximum growth rates (*r*_max_). Secondly, single‐cell analysis of the ciliates' food vacuole content yielded similar estimates to those based on the analysis of ciliate population dynamics, i.e. the change in ciliate cell numbers.

We applied optical methods (i.e. microscopy and imaging flow cytometry) to investigate the ciliates' response to starvation. While light microscopy has a long tradition, the latter is a relatively novel approach in protistological research that we have used, for the first time, to monitor a ciliate population exposed to starvation. We demonstrated that imaging microscopy has a high potential to estimate heterotrophic protists' nutritional status and other physiological characteristics.

In the near future, transcriptomic gene expression analyses of key enzymes of ingestion and digestion of ciliates and other phagotrophic protists (Caron et al., 2017; Zou et al., 2020) may complement the optical methods for ciliates and other heterotrophic protists. Like imaging flow cytometry, single‐cell transcriptomics can potentially estimate the predator population's phenotypic plasticity upon starvation.

## FUNDING INFORMATION

Funding for this work was provided by the Austrian Science Fund (FWF, Project P 32714‐B), awarded to TW.
